# 

**Published:** 1886-11-06

**Authors:** 


### CONTENTS.

**Medical Students, their Schools and Schoolmasters** - 87

**Discharged. Not where to Lay his Head.** By George Fleming. Chapters VII, VIII, IX - 88

**Old-Fashioned Pauper Infirmaries** - 90

**A Noble Profession: Nursing the Sick.**—Nursing at St. Mary's Hospital. By the Secretary, Mr. P. Michelli - 92

**Words of Consolation.**—VI. The Privilege of Suffering - 93

**Poetry.**—A Plea for the Dumb Animals - 93

**Notes and News.**—H.R.H. the Duke of Cambridge.—Miscellaneous.—Fever Cases in the Metropolis.—Affairs at the Western Asylum.—Trade Societies' Demonstrations.—Friendly Societies and the Hospitals.—The Dudley Dispensary.—Toys for Poor Children.—Vacancies.—The Prince's Cinderellas.—Royal Aberdeen Infirmary.—Taunton and Somerset Hospital.—"The Darlington Hospital Scandal," etc. - 94

**The Out-Patient Registration Fee System.** By J. C. Steele, M.D. - 97

**Annotations.**—Residential Accommodation for Medical Students.—Infected Cabs.—Lodging-House Keepers and their Consciences - 98

**Till the Doctor Comes.**—Chapter III.—Diseases of Children - 99

**Flowers, Ferns, and Ward Decorations** - 100

**The Editor's Letter-Box.**—Homes on the Riviera.—Nurses' Food and Feeding.—Milk and Alcohol in Children's Hospitals, etc. - 101

**The Literary Prize.**—Conditions and Results - 102

**The Hospital Charity Box.**—Prizes and Results - 102

**The Children's Corner.**—Puzzles, Answers, etc. - 102

**In- and Out-Patients' Hospital Diary** - 103

